# Application of arbitrary and individualized load quantification strategies over the weekly microcycle in professional soccer players

**DOI:** 10.5114/biolsport.2024.129481

**Published:** 2023-07-21

**Authors:** Alexis Padrón-Cabo, David Solleiro-Duran, Miguel Lorenzo-Martínez, Fabio Y. Nakamura, Miguel Ángel Campos-Vázquez, Ezequiel Rey

**Affiliations:** 1 University of A Coruña, Department of Physical and Sports Education, Faculty of Sport Sciences and Physical Education, A Coruña, Spain; 2 University of Vigo, Faculty of Education and Sport Sciences, Pontevedra, Spain; 3 Research Center in Sports Sciences, Health Sciences and Human Development (CIDESD), University of Maia, 4475-690 Maia, Portugal; 4 Department of Sport Science, Pablo de Olavide University, Sevilla, Spain

**Keywords:** Global Positioning System, Workload monitoring, Peak velocity, 30–15 Intermittent Fitness Test

## Abstract

The aims of this study were to: (a) determine the differences in external load quantification between arbitrary and individual speed thresholds over the weekly microcycle in professional soccer players, and (b) analyse the association between internal load and different external load quantification strategies (ELQSs). Ten professional outfield players were monitored during training sessions and official matches using 10 Hz GPS devices over a 6-week in-season period. The absolute and relative (“R” before the distance category) distances covered were calculated for the following external load variables: medium-intensity running distance (MIR), high-intensity running (HIR), sprint distance (SD), and very high-intensity running (VHIR). Individualized thresholds were determined based on maximal sprinting speed (MSS) and the last speed achieved during the 30–15 Intermittent Fitness Test (V_IFT_) of each player. In terms of match-day workload, significant differences (*p* < 0.05) were observed between arbitrary and individualized strategies (i.e., MSS and V_IFT_) for the distance covered in MIR, HIR, SD, VHIR, RHIR, RSD, and RVHIR. The MSS strategy compared to arbitrary thresholds revealed significant differences (*p* < 0.05) for distance covered in HIR, RHIR, and VHIR during all training sessions. The present results showed that arbitrary thresholds lead to underestimation of external load absolute and relative metrics compared to the MSS strategy throughout the microcycle. The V_IFT_ strategy mainly revealed differences in external load quantification regarding MD compared to arbitrary thresholds. Individualized speed threshold strategies did not achieve better associations with internal load measures in comparison with arbitrary thresholds in professional soccer players.

## INTRODUCTION

Time-motion analysis and physiological monitoring have frequently been used to analyse the movement patterns and workloads imposed by the match play and training sessions in soccer [1]. According to previous literature, the workload can be differentiated into internal and external loads [2]. In that sense, Global Positioning System (GPS) technology is commonly used to monitor and record external load in professional soccer [3]. The appropriate management of daily external load has been considered a central issue in optimizing microcycle programming [4, 5]. Recently, an expert panel belonging to teams from Big-5 European football leagues stated that high-intensity running (HIR) and sprinting-focused exercises were perceived as the most effective strategies for preventing lower-limb injuries [6]. Additionally, the ability to reproduce high-intensity actions during matches is a key element in gaining advantages during offensive and defensive tactical situations [7, 8]. In this regard, Beato et al. [9] established that implementing HIR and sprint training has a pivotal role in developing or maintaining the intermittent ability to execute high-intensity actions during a soccer match. Therefore, one might state that accurate monitoring and management of HIR and sprinting exposure across microcycles reduce the likelihood of non-contact injuries and optimize the ability to perform high-intensity actions during competitive scenarios [9, 10].

Despite the recognized relevance of external load monitoring, the player’s locomotor demands are generally analysed using different running thresholds determined arbitrarily [3, 11]. Concerning HIR and sprinting, the implementation of the arbitrary threshold may mask the player’s real efforts, leading to an inadequate interpretation of actual metabolic and neuromuscular demands during training or competition [12, 13]. In order to individualize speed thresholds, researchers have used different methods based on physiological laboratory and field tests [11, 12, 14, 15, 16]. However, the implementation of physiological tests comprising continuous or linear movements does not reflect the ability of soccer players to perform changes of direction and high-intensity actions [13]. Conversely, the action-specific field tests could be recognized as ecological, time-efficient, and cost-effective alternatives to determine the individual speed thresholds [17, 18]. Consequently, it seems necessary to develop field methods for quantifying the external load considering the individual physical capacity of soccer players [9].

Recent studies have established the possibility of individualizing speed thresholds by maximal sprinting speed (MSS) and functional fitness tests (e.g., the 30–15 Intermittent Fitness Test) [9, 12, 16, 19]. Regarding MSS, Kyprianou et al. [20] pointed out that it is a key metric for external load individualization since exposure to sprint (ranging from > 85% to > 95% of MSS) during training (i.e., match day minus 2) might be related to a reduction in hamstring injuries during matches [21]. Likewise, Massard et al. [22] showed that MSS could be easily defined with GPS data due to its strong association with the 40-m sprint test. In addition, to better prescribe and monitor HIR-related metrics, other studies have individualized the speed zones using the velocity reached during the last successful stage of the 30–15 Intermittent Fitness Test (V_IFT_) [12, 16]. During the last few years, this test has been widely used to assess intermittent physiological capacities (i.e., maximal oxygen uptake (VO_2max_), change of direction and inter-effort recovery ability, and to prescribe high-intensity interval training (HIIT) in soccer players [23]. In fact, the drills based on V_IFT_ have been recommended to provide a dose of weekly HIR in soccer players, especially for non-starter players [24]. Therefore, individualization of speed thresholds based on MSS and V_IFT_ could provide a more practical approach for training monitoring and for replicating match-equivalent HIR and sprinting load, while accounting for individual fitness levels.

To the best of our knowledge, there are limited previous comparisons of external load resulting from arbitrary and individualized speed thresholds within the typical microcycle in professional soccer players [25, 26]. Furthermore, no previous research has analysed possible discrepancies between arbitrary and individual speed thresholds, as determined using V_IFT_. Therefore, the aim of this study was to determine the differences in external load quantification between arbitrary and individual speed thresholds based on MSS and V_IFT_ over the weekly microcycle in professional soccer players. Additionally, the secondary aim was to analyse the association between internal load (i.e., rating of perceived exertion (RPE) and session-RPE (sRPE) and distance covered in HIR and sprinting depending on the external load quantification strategy (ELQS). In line with previous research [16, 17], it is hypothesized that the arbitrary thresholds underestimate or overestimate the distance covered at HIR and sprinting depending on the player’s physical fitness status.

## MATERIALS AND METHODS

### Participants

A total of 10 male outfield Spanish professional soccer players (mean ± SD; age = 26.52 ± 4.25 years; height = 178.0 ± 6.36 cm; body mass = 73.47 ± 3.24 kg) who belonged to the same squad during the season 2021–2022 participated in this study. According to the positional role, the distribution of players was as follows: central defenders (2), fullbacks (2), midfielders (4), and forwards (2). All participants had experience at the professional soccer level from a minimum of one year to a maximum of nine years. Players regularly trained 5 times per week and training sessions had a duration 45 to 80 min (average duration = 62.8 ± 9.66 min) depending on the day of the microcycle. In addition, the team competed in one match per week. The inclusion criteria were that the players completed 80% of all training sessions and played at least one full match during the monitoring period. Moreover, the goalkeepers were excluded from data collection due to the differences in external load demands. The research protocol was approved by the investigation review committee (code 10-0721). The study fulfilled the ethical requirements and principles of the Declaration of Helsinki.

### Experimental approach to the problem

A retrospective longitudinal observational research study was conducted on a Spanish professional soccer team during the 2021–2022 end-season period. External load data from 6 competitive microcycles were collected. Throughout the data collection period, players completed 3–5 on-field training sessions and one official match per week. In line with Akenhead et al. [27], the days of the microcycle were categorized in relation to the match day (MD): 4 days before the match (MD-4), 3 days before the match (MD-3), 2 days before the match (MD-2), and 1 day before the match (MD-1). The training contents of each session are described in Table 1. Only data from the principal training sessions of the team were recorded. Records from regeneration and compensatory sessions (i.e., 1 day after match sessions) were excluded. In reference to match analysis, only the files of players who participated for at least 80 min were retained for analyses [28]. The external load of each session and match was calculated using standardized (arbitrary), and individualized speed thresholds. Specifically, the individualized speed thresholds were calculated from the MSS of each player and the speed reached at the end of the 30–15 Intermittent Fitness Test (V_IFT_). In total, 585 observations from 20 training sessions and 6 official matches were registered for the subsequent statistical analysis.

**TABLE 1:** Training contents for each microcycle session.

Day	Description
**MD-4**	Strength training, small possession and position games, small-sided games, and repeat sprint training.
**MD-3**	Rondos, tactical drills, pressing tasks, physical-technical circuits based on V_IFT_, medium-sided games, and partial time simulated 11 vs 11 matches.
**MD-2**	Rondos, control and passing tasks, and tactical drills.
**MD-1**	Activation drills, rondos, 11 vs 11 games (half pitch), and review of tactical keys regarding the match.
**MD**	Match day

MD: match day; MD-5: 5 days before match; MD-4: 4 days before match; MD-3: 3 days before match; MD-2: 2 days before match; MD-1: 1 day before match.

### Procedures

**FIG. 1:**
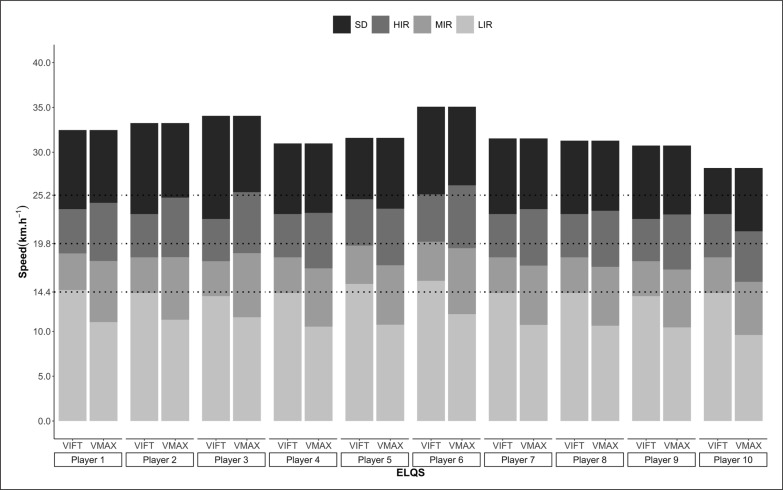
Displays the arbitrary and individualized (i.e., MSS and V_IFT_ strategies) speed thresholds for each player.

During the intervention period, the external load of each session and match was gathered using a portable 10-Hz GPS device (Playertek, Catapult Innovations, Melbourne, Australia). According to Scott et al. [29], GPS units with a sampling rate of 10-Hz are the most valid and reliable for external load analysis in team sports. In order to avoid inter-unit variability error, all players used the same GPS unit during training sessions and matches. To increase the reliability of data collection, the GPS units were activated 15 minutes prior to each training session or match in an open area to acquire a minimum of 6 satellites avoiding weak connections and ensuring data quality [3, 30, 31]. Subsequently, players wore a custom-made vest with a small pocket between the shoulder blades, where the GPS unit was placed. After each session, the data were transferred using the manufacturer’s software in order to calculate standardized and individualized external load variables. In this regard, each session was processed three times, applying different speed thresholds based on standardized, MSS, and V_IFT_ external load quantification strategies (Figure 1). Regardless of ELQS, all variables were analysed in terms of volume (i.e., absolute distance covered in metres) and intensity (i.e., distance covered relative to session or match duration), considering each speed threshold [4, 5]. In line with previous research, external load data were analysed from 6 competitive microcycles in order to reduce the effects of physical performance variations within the competitive season in MSS and V_IFT_ strategies [16, 32].

The arbitrary speed thresholds were established in accordance with previous research [4, 30]: medium-intensity running distance (MIR; 14.4–19.79 km·h^−1^), high-intensity running (HIR; 19.8–25.1 km·h^−1^), and sprint distance (SD; > 25.2 km · h^−1^). In addition, the distance covered above 19.8 km · h^−1^ was categorized as very high-intensity running (VHIR).

The 30–15 Intermittent Fitness Test (30–15_IFT_) was performed as previously described [33, 34]. The 30–15_IFT_ consisted of 30-s shuttle runs interspersed with 15-s passive recovery periods. The initial running velocity was set at 8 km · h^−1^ for the first 30-s shuttle run and increased by 0.5 km · h^−1^ until the end of the protocol. The players ran back and forth between two lines set 40 m apart. The pace of shuttle runs was governed by audio beeps, allowing players to adjust their running velocity to each stage. During the 15-s recovery period, the players walked forward until the nearest 3-m zone located in the middle or at one end of the running area, awaiting the beginning of the next running stage. Players were instructed to complete as many stages as possible. The test was considered finished when players were unable to maintain the running pace established by audio beeps or when they were unable to reach the 3-m zone around each line at the moment of the audio signal, 3 consecutive times. The velocity reached during the last successful stage of 30–15_IFT_ was used for individualizing the speed threshold of each player. Previous research determined high validity and reliability for the 30–15_IFT_ outcomes in team sports athletes [35, 36]. Players presented an average V_IFT_ of 21.30 ± 0.82 km · h^−1^. Individualized speed thresholds based on the V_IFT_ strategy were established as follows [16, 37]: MIR (68–86.99% V_IFT_), HIR (86.99–110% V_IFT_), and SD (> 110% V_IFT_). For this strategy, the distance covered above 86.99% V_IFT_ was classified as VHIR. All players conducted the 30–15IFT during the week before the data collection period.

Regarding the MSS strategy, the peak speed was retrospectively established by analysing the speed obtained in training sessions and matches throughout the competitive season [38]. Recent research has established that 10 Hz GPS technology can provide valid and reliable related to a player’s peak speed [22]. Likewise, the data referring to sprint and repeated sprint ability training were included to establish peak speed. The average peak speed was 31.90±1.92 km·h^−1^. In accordance with Murray et al. [15], the MSS thresholds were categorized as follows: MIR (34–54.99% MSS), HIR (55–74.99% MSS), and SD (> 75% MSS). For this strategy, the distance covered above 54.99% MSS was classified as VHIR.

The 0–10 Foster’s RPE was registered to quantify the internal load of each player [39]. All participants recorded individually their RPE 30 minutes after the end of the training sessions to avoid potential bias [40]. Players were familiar with the use of the RPE scale because they used it as a part of their regular training routine. Additionally, the S-RPE was calculated by multiplying the RPE score provided for each player during training sessions by the training duration.

### Statistical analyses

The results were presented as means and standard deviations (mean ± SD). All analyses were performed using the statistical software R version 4.2.1 (R Core Team, 2020) for Macintosh. A linear mixed model was adjusted using the R package “lm4” [41] to analyse the differences in external load quantification strategies (i.e., “standard” arbitrary strategy, and individualization ones using V_IFT_ and MSS as references) for the different speed thresholds (i.e., MIR, HIR, SD), according to the microcycle session (i.e., MD-4, MD-3, MD-2, MD-1, MD). Player identity was determined as a random effect to account for the repeated measures. In this regard, the following model was adjusted for all running variables (*y*):

y=ELQS⋅Session+(1|Player ID)

For each model, the assumptions of homogeneity and normal distribution of the residuals were checked. The models’ residuals fulfilled the assumption of homogeneity and normal distribution. The R package “*emmeans*” [42] was used to perform pairwise comparisons via the Bonferroni post-hoc test between external load quantification strategies. Additionally, effect sizes were determined using Cohen’s *d* with the following formula: *d* = (M_2_ – M_1_)/SD_pooled_. According to Hopkins et al. [43], effect sizes were classified as trivial (0.0–0.2), small (0.2–0.6), moderate (0.6– 1.2), large (1.2–2.0), and very large (> 2.0). Repeated measures correlations between RPE and external load quantification strategies using the different speed thresholds were examined using the R package “*rmcorr*” [44]. The magnitude of correlation was categorized using the following criteria [39]: < 0.10, trivial; 0.10 to 0.29, small; 0.30 to 0.49, moderate; 0.50 to 0.69, large; 0.70 to 0.89, very large; and > 0.90, nearly perfect. The significance value was established for all statistical analyses at *p* < 0.05.

## RESULTS

### Effects of external load quantification strategies on the training and match day’s workload.

Table 2 displays the absolute and relative (“R” before the distance category) values of workload as well as the significant differences observed between ELQSs during a weekly microcycle. In terms of MD workload, the MSS and V_IFT_ strategies presented significantly greater distance covered in HIR (ES: 1.49 and 0.64, large and moderate), SD (ES: 0.63 and 0.97, moderate), VHIR (ES: 1.35 and 0.85, large and moderate), RHIR (ES: 1.38 and 0.60, large and moderate), RSD (ES: 0.60 and 0.93, moderate), and RVHIR (ES: 1.26 and 0.81, large and moderate) than the arbitrary strategy. For the speed threshold of MIR, the MSS (ES: 3.76, very large) and arbitrary (ES: 0.95, moderate) strategies displayed significantly greater distance covered in comparison with V_IFT_. In addition, the MSS strategy displayed significantly greater distances in HIR (ES: 1.11, moderate), VHIR (ES: 0.75, moderate), RHIR (1.01, moderate), and RVHIR (ES: 0.70, moderate) compared to V_IFT_. Regarding MD-4, the MSS strategy resulted in significantly greater distance in MIR (ES: 3.82, very large), HIR (ES: 1.13, moderate), SD (ES: 0.25, small), VHIR (ES: 0.75, moderate), RMIR (ES: 3.21, very large), RHIR (ES: 1.18, moderate), and RVHIR (ES: 0.84, moderate) compared to the arbitrary strategy. Additionally, the MSS strategy exhibited a greater distance covered in MIR (ES: 4.47, very large) and RMIR (ES: 3.68, very large) than V_IFT_, while V_IFT_ showed a significantly greater distance covered in RSD (ES: 0.38, small) than the arbitrary strategy. In reference to MD-3, the workload quantified with the MSS strategy resulted in significantly greater MIR (ES: 2.75, very large), HIR (ES: 1.52, large), SD (ES: 0.80, moderate), VHIR (ES: 0.81, moderate), RMIR (ES: 2.50, very large), RHIR (ES: 1.56, large), and RVHIR (ES: 1.51, large) distance compared to the arbitrary strategy. In addition, the MSS presented higher values in MIR (ES: 3.20, very large), HIR (ES: 1.10, moderate), VHIR (ES: 0.74, moderate), RMIR (ES: 2.89, very large), RHIR (ES: 1.16, moderate), and RVHIR (ES: 0.78, moderate) compared to V_IFT_, while V_IFT_ revealed greater distance covered in SD (ES: 0.98, moderate) and RSD (ES: 1.00, moderate) than the arbitrary strategy. For MD-2 and MD-1, the MSS external load quantification strategy displayed significantly greater distance covered in MIR (ES: 2.58 and 2.45, very large), HIR (ES: 1.00 and 1.52, moderate and large), RMIR (ES: 3.27 and 2.45, very large), RHIR (ES: 1.19 and 1.51, moderate and large), and RVHIR (ES: 1.23 and 1.45, large) in comparison with the arbitrary strategy. However, the MSS strategy exhibited significantly greater distance in VHIR (ES: 1.05, moderate) on MD-2 but not on MD-1 (p > 0.05). Additionally, the V_IFT_ strategy revealed higher values in RSD (ES: 1.21, large) than the arbitrary strategy on MD-2.

**TABLE 2:** Differences (mean ± SD) in the distance covered in different speed zones according to arbitrary and individualized thresholds during competitive microcycle.

Variable	ELQS	MD-4 (mean ± SD)	MD-3 (mean ± SD)	MD-2 (mean ± SD)	MD-1 (mean ± SD)	MD (mean ± SD)
MIR (m)	ARB	381.30 ± 82.95	622.64 ± 131.69	256.74 ± 77.99	331.71 ± 94.45	1s754.97 ± 415.21
	V_IFT_	318.69 ± 67.37	508.70 ± 84.63	255.74 ± 152.26	273.94 ± 73.11	1415.87 ± 284.88^*^
	MSS	878.40 ± 163.75^*#^	1470.92 ± 415.48^*#^	610.24 ± 177.21^*#^	829.76 ± 270.99^*#^	3286.87 ± 642.64^*#^

HIR (m)	ARB	122.56 ± 69.51	223.71 ± 94.16	119.50 ± 53.13	85.67 ± 34.31	650.47 ± 199.02
	V_IFT_	148.26 ± 64.98	268.05 ± 86.18	156.04 ± 78.31	117.14 ± 40.68	771.84 ± 180.10^*^
	MSS	211.00 ± 85.32^†^	385.23 ± 122.97^*#^	214.29 ± 121.99^†^	167.51 ± 67.78^†^	1097.43 ± 373.55^*#^

SD (m)	ARB	68.45 ± 87.06	44.80 ± 33.39	19.27 ± 16.65	8.69 ± 10.04	194.25 ± 118.50
	V_IFT_	105.42 ± 118.93	89.60 ± 55.22^†^	49.35 ± 31.99	23.30 ± 18.35	312.09 ± 125.14^*^
	MSS	92.74 ± 107.81	76.18 ± 43.50	38.63 ± 27.59	17.71 ± 16.70	265.15 ± 104.45^*◆^

VHIR	ARB	191.01 ± 145.22	268.52 ± 114.43	138.77 ± 60.69	94.37 ± 38.44	844.71 ± 294.62
	V_IFT_	253.69 ± 168.14	357.65 ± 125.69	205.40 ± 100.29	140.44 ± 48.99	1083.94 ± 264.41^*^
	MSS	308.05 ± 164.41^†^	461.42 ± 151.97^*◆^	252.92 ± 141.59^†^	185.23 ± 76.74	1362.59 ± 453.49^*#^

RMIR (m/min)	ARB	5.76 ± 1.26	8.54 ± 1.86	4.76 ± 1.07	5.58 ± 1.57	18.57 ± 4.91
	V_IFT_	4.82 ± 1.03	6.98 ± 1.23	4.65 ± 2.32	4.61 ± 1.22	14.97 ± 3.44^*^
	MSS	13.43 ± 3.14^*#^	20.30 ± 6.39^*#^	11.40 ± 2.66^*#^	14.00 ± 4.76^*#^	34.58 ± 6.98^*#^

RHIR (m/min)	ARB	1.81 ± 1.01	3.04 ± 1.19	2.18 ± 0.78	1.44 ± 0.58	6.87 ± 2.20
	V_IFT_	2.20 ± 0.93	3.65 ± 1.09	2.83 ± 1.16	1.98 ± 0.70	8.16 ± 2.08^*^
	MSS	3.16 ± 1.27^*^	5.26 ± 1.63^*#^	3.87 ± 1.85^*◆^	2.83 ± 1.17^*^	11.58 ± 4.29^*#^

RSD (m/min)	ARB	0.94 ± 1.15	0.61 ± 0.45	0.36 ± 0.31	0.14 ± 0.17	2.06 ± 1.30
	V_IFT_	1.47 ± 1.60^†^	1.22 ± 0.74^†^	0.90 ± 0.55^†^	0.39 ± 0.31	3.29 ± 1.34^*^
	MSS	1.29 ± 1.44	1.04 ± 0.58	0.71 ± 0.47	0.30 ± 0.28	2.79 ± 1.14^*^

RVHIR (m/min)	ARB	2.75 ± 1.98	3.65 ± 1.46	2.54 ± 0.92	1.60 ± 0.66	8.93 ± 3.25
	V_IFT_	3.67 ± 2.26	4.88 ± 1.62^†^	3.74 ± 1.50	2.38 ± 0.86	11.44 ± 2.95^*^
	MSS	4.52 ± 2.22^*^	6.30 ± 1.99^*◆^	4.58 ± 2.15^*^	3.13 ± 1.33^*^	14.38 ± 5.18^*#^

Abbreviations: MIR: medium-intensity running distance; HIR: high-intensity running; SD: sprint distance; VHIR: very high-intensity running; RMIR: relative medium-intensity running distance; RHIR: relative high-intensity running distance; RSD: relative sprint distance; RVHIR: relative very high-intensity running; ARB; arbitrary running threshold; MSS: individualized threshold based on maximal sprint speed; V_IFT_: individualized threshold based on final speed reached at the end of 30–15 Intermittent Fitness Test; MD: match day; MD-5: 5 days before match; MD-4: 4 days before match; MD-3: 3 days before match; MD-2: 2 days before match; MD-1: 1 day before match; ELQS: external load quantification strategy.

* Significant differences (p < 0.01) with arbitrary external load quantification strategy.

# Significant differences (p < 0.01) with V_IFT_ external load quantification strategy.

‡Significant differences (p < 0.01) with MSS external load quantification strategy.

† Significant differences (p < 0.05) with arbitrary external load quantification strategy.

◆ Significant differences (p < 0.05) with V_IFT_ external load quantification strategy.

### Relationship between RPE and external load according to quantification strategy.

Table 3 presents repeated measures correlation coefficients for RPE and sRPE with workload calculated through different quantification strategies. The sRPE was associated with some absolute values of workload. VHIR showed moderate associations (r_rm_ = 0.59 to 0.63) with sRPE regardless of the strategy used. Similarly, the MIR had moderate correlations with sRPE for arbitrary (r_rm_ = 0.68) and V_IFT_ strategies (r_rm_ = 0.59), while MSS presented a small correlation (r_rm_ = 0.47). Additionally, the RPE was associated with some relative values. For RVHIR, all external load quantification strategies showed small associations with RPE (r_rm_ = 0.38 to 0.41). There was a moderate association between RMIR and RPE in the arbitrary strategy (r_rm_ = 0.51), while the correlations were classified as small-to-trivial for V_IFT_ and MSS strategies, respectively.

**TABLE 3:** Within-player correlation coefficients for the relationship between RPE, sRPE and external load according to different methods.

		Arb			VIFT			MSS		

		*r*	95% CI	Magnitude	*r*	95% CI	Magnitude	*r*	95% CI	Magnitude
sRPE	MIR (m)	0.68**	0.58–0.75	Moderate	0.59**	0.48–0.69	Moderate	0.47**	0.33–0.59	Small
	VHIR (m)	0.59**	0.47–0.68	Moderate	0.60**	0.48–0.69	Moderate	0.63**	0.51–0.71	Moderate

RPE	RMIR (m/min)	0.51**	0.38–0.62	Moderate	0.38**	0.23–0.51	Small	0.16*	0.01–0.31	Trivial
	RVHIR (m/min)	0.39**	0.25–0.52	Small	0.38**	0.23–0.51	Small	0.41**	0.27–0.53	Small

Abbreviations: ARB; arbitrary running threshold; MSS: individualized threshold based on maximal sprint speed; V_IFT_: individualized threshold based on final speed reached at the end of 30–15 Intermittent Fitness Test; MIR: medium-intensity running distance; VHIR: high-intensity running; RMIR: relative medium-intensity running distance; RVHIR: relative very high-intensity running

** p-value < 0.01;

* p-value < 0.05.

## DISCUSSION

This study analysed the differences in external load quantification between arbitrary and individualized thresholds based on MSS and V_IFT_ across the microcycle, and associations between internal load and distance covered in MIR and HIR depending on the ELQS in professional soccer players. To the authors’ knowledge, this is the first study to examine the differences in external load quantification using individualized thresholds based on the V_IFT_ and MSS across the microcycle days. The main findings of the study are that (a) arbitrary thresholds underestimated the distance covered in all absolute and relative speed thresholds compared to the MSS external load strategy, whilst (b) the V_IFT_ strategy showed similar values to the arbitrary threshold for all training sessions, although underestimating the external load for all variables in MD, except for MIR and RMIR. Additionally, the associations between internal and external load metrics (i.e., MIR and HIR) showed similar relationships independently of the ELQS used.

From a physical performance perspective, the distance covered in HIR has been considered a crucial metric for attaining successful participation in competitive soccer scenarios [9]. Moreover, several studies determined the relationship between distance covered in HIR and injury occurrence in high-intensity intermittent team sports [10, 45]. Because of inappropriate HIR load monitoring, the coaches and practitioners could miss the possibility of providing an efficient physiological stimulus and reducing the likelihood of non-contact injuries in professional soccer [6, 9]. In the current study, our results revealed that distance covered in HIR, RHIR, and VHIR was higher in all training sessions and MD (~50–60%) when speed zones were individualized using MSS compared to arbitrary thresholds. Similarly, Hunter et al. [11] observed differences of 39% to 61% in total high-speed running and total very high-speed running when adjusting speed bands based on MSS compared to arbitrary thresholds in U18 elite soccer players. More recently, Rago et al. [26] analysed the differences in external load quantification in professional soccer players using individualized speed thresholds considering maximal aerobic speed (MAS), ASR, and MSS. In line with our results, these authors found likely moderate differences (ES: 0.86) in the distance covered in HIR when speed thresholds were individualized in comparison with arbitrary thresholds over the microcycle. Conversely, in reference to the V_IFT_ strategy, the distance covered in HIR solely showed a significantly greater distance covered in HIR, RHIR, and VHIR in MD. Thus, the lack of differences observed between the V_IFT_ strategy and arbitrary thresholds across the microcycle could be explained by a reduced HIR bandwidth compared to the MSS strategy. However, implementing the V_IFT_ strategy could contribute to better monitoring and management of the HIR weekly dose in professional soccer players, taking into account their metabolic characteristics to reproduce high-intensity intermittent efforts, and avoid exposure to large and abrupt spikes in HIR distance [9, 46].

Sprinting is considered a critical element due to its potential to concurrently increase performance and reduce injury risk in team sports athletes [6, 47, 48]. In fact, hamstring injury incidence could be reduced when elite soccer players were exposed to running bouts at near-to-maximal speed during training sessions [21, 49]. However, the implementation of arbitrary thresholds (i.e., > 25 km · h^−1^) to monitor sprint weekly dose could lead to load error because of the lack of specificity in relation to the player’s MSS [3, 13, 50]. In this study, the results showed that arbitrary thresholds underestimated the SD and RSD compared to MSS and V_IFT_ strategies on MD. Both individualized ELQS presented higher values in the distances covered in SD and RSD according to the player’s individual locomotor profile. However, the V_IFT_ strategy could lead to an error (i.e., mainly overestimation) in SD quantification. Specifically, the V_IFT_ strategy would not allow proper monitoring of speeds considered protective (i.e., 85–95% MSS), when soccer players display a low value of V_IFT_ during 30–15IFT and their MSS values are high [21, 47]. In this regard, the SD thresholds individualized based on MSS could ensure that players are monitored regarding their own sprint ability, avoiding under- or overestimation in SD exposure [51]. Further research is needed to elucidate the benefits of monitoring SD using different percentages of MSS (i.e., 85–95%) over the microcycle, and its association with injury risk.

To obtain an accurate dose-response relationship, it is necessary to monitor internal and external loads during training sessions or matches in team sports [2, 52]. Currently, there is limited evidence from analyses of the associations between speed thresholds individualized in accordance with players’ physical characteristics and fitness and internal load in soccer players [53, 54]. Our results showed small to moderate within-individual correlations between sRPE and MIR, and moderate correlations with the VHIR metric for the different ELQSs. Regarding RPE, our results revealed small correlations for RVHIR in all ELQSs, but small to moderate in the RMIR metric for arbitrary and V_IFT_ strategies. Previously, Scott et al. [53] examined the associations between speed thresholds individualized according to different physiological measures (i.e., MSS, MAS heart rate deflection point, or final speed achieved during the Yo-Yo intermittent recovery test) and internal load (i.e., RPE, TRIMP (TRaining IMPulse), minutes spent above 80% maximal heart rate) in international female soccer players. In accordance with our data, these authors found that individualized speed thresholds did not show stronger associations with internal load variables than arbitrary thresholds. Likewise, Sparks et al. [54] reported that non-significant correlations (*r* = 0.10, trivial) were found between HIR individualized by MSS and heart rate responses in university-level soccer players. In the present study, it is noteworthy that only RPE and sRPE were analysed as a reflection of the player’s internal load. Perhaps the presence of similar associations between arbitrary and individualized time-motion analysis strategies with the internal load measures could be explained by the low variability of RPE response (CV: ~5%) and the high variability in HIR and SD across the microcycle (CV: > 80%) and competition (CV range: 19.8–53%) in soccer [4, 55, 56, 57]. Future studies should consider the inclusion of more internal load variables (e.g., heart rate, blood lactate, perceived tissue damage, self-reported wellness measures) in order to investigate the relationships with external load individualized speed thresholds in professional soccer players.

The findings of this research should be interpreted with caution due to some specific limitations. Firstly, the sample is composed of only one professional team. The results could not be generalized to other soccer backgrounds, leagues, or competitive levels (i.e., amateur or semi-professional squads), due to the differences in competitive demands [58]. Secondly, we examined a total of 6 microcycles, which could be considered a relatively small sample size. However, this sample size was similar to other studies that analysed the differences between different ELQSs [17, 26]. Thirdly, the physiological cut-off points of 30–15IFT were determined in line with previous research [16, 59] due to the complexity of applying laboratory assessments in large team sports squads. Lastly, the individualized speed thresholds were established according to specific physical attributes (i.e., MSS, V_IFT_). In this regard, it has been suggested [11] that a combined approach (i.e., two different physical measures) could contribute to establishing locomotor profiles accurately, improving the quantification of external load. In line with Clemente et al. [25], further studies should attempt to implement a combined approach with V_IFT_ and MSS in order to provide better management of the dose-response relationship in soccer players.

## CONCLUSIONS

Workload monitoring is a relevant piece of the puzzle to appropriately programme training sessions, recovery strategies, and training drills during the microcycle. The application of arbitrary thresholds might lead to an external load quantification misconception by soccer coaches and sports scientists. This study showed that arbitrary thresholds lead to underestimation of external load absolute (i.e., MIR, HIR, and VHIR) and relative (i.e., RMIR, RHIR, and RVHIR) metrics compared to the MSS strategy throughout the microcycle. The V_IFT_ strategy mainly revealed differences in external load quantification regarding MD compared to arbitrary thresholds. Likewise, both individualized strategies led to different results in terms of external load quantification across microcycle days. Finally, the individualized speed threshold strategies did not achieve better associations with internal load measures (i.e., RPE, sRPE) in comparison with arbitrary thresholds in professional soccer players.
